# Supplementary material for: Functional Genetic Screen to Identify Interneurons Governing Behaviorally Distinct Aspects of Drosophila Larval Motor Programs
Source: G3 (Bethesda). 2016 May 6;6(7):2023–31. doi: 10.1534/g3.116.028472 (PMC4938655; doi:10.1534/g3.116.028472)
Supplement: Supplemental Material [file supp_6_7_2023__index.html]

Functional Genetic Screen to Identify Interneurons Governing Behaviorally Distinct Aspects of Drosophila Larval Motor Programs — Functional Genetic Screen to Identify Interneurons Governing Behaviorally Distinct Aspects of Drosophila Larval Motor Programs — Supplemental Material 

# Functional Genetic Screen to Identify Interneurons Governing Behaviorally Distinct Aspects of *Drosophila* Larval Motor Programs

## Supplemental Material for Clark *et al.*, 2016

**Files in this Data Supplement:**

- File S1 - Matlab scripts for automated analysis of the speed of larval locomotion. (.zip, 29 KB)
- Table S1 - Table of larval locomotion speeds of all Rubin Gal4 lines screened against *UAS-dTrpA1*. (.xlsx, 83 KB)
